# Supplementary material for: Autoimmunity against Nucleus Ambiguous Is Putatively Possible in Both Long-COVID-19 and Vaccinated Subjects: Scientific Evidence and Working Hypothesis
Source: Biology (Basel). 2024 May 21;13(6):359. doi: 10.3390/biology13060359 (PMC11200469; doi:10.3390/biology13060359)
Supplement: Supplementary file 1 [file biology-13-00359-s001.zip › biology-2985859-supplementary.pdf]

Supplementary Materials

Brief Report

Autoimmunity against Nucleus Ambiguous Is Putatively Possible in Both Long-COVID 19 and Vaccinated Subjects: Scientific Evidence and Working Hypothesis

Original blots

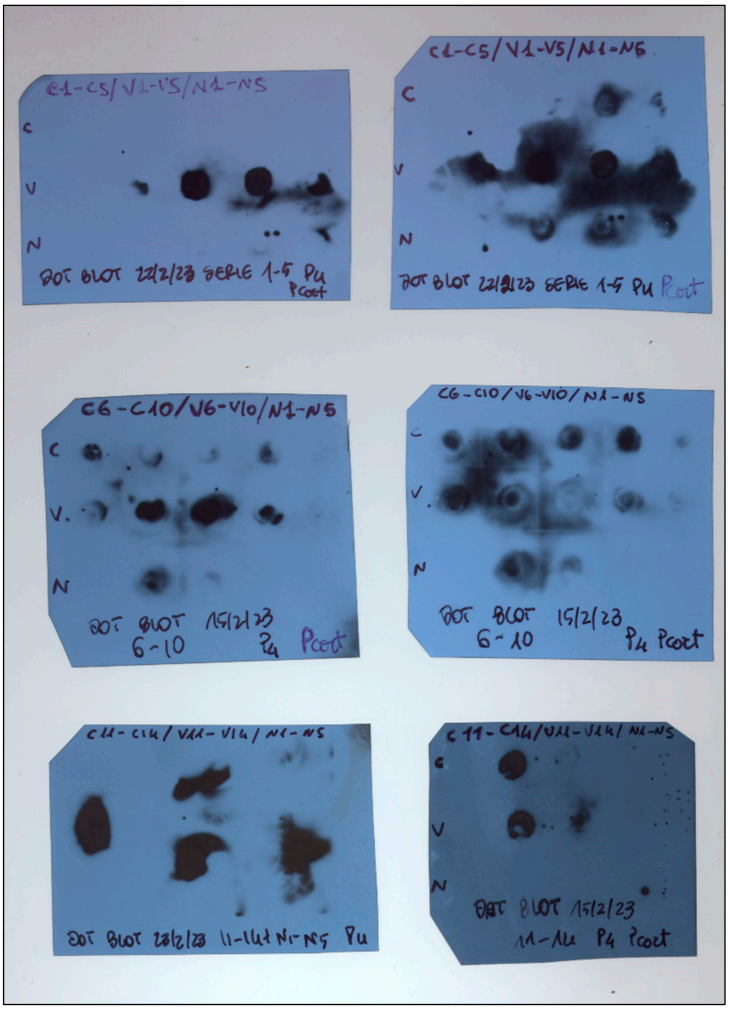

Figure S1. Original blots for Pcort. The original image corresponding to the figure in the main article is a cropping containing the sample C6-V6-N1.

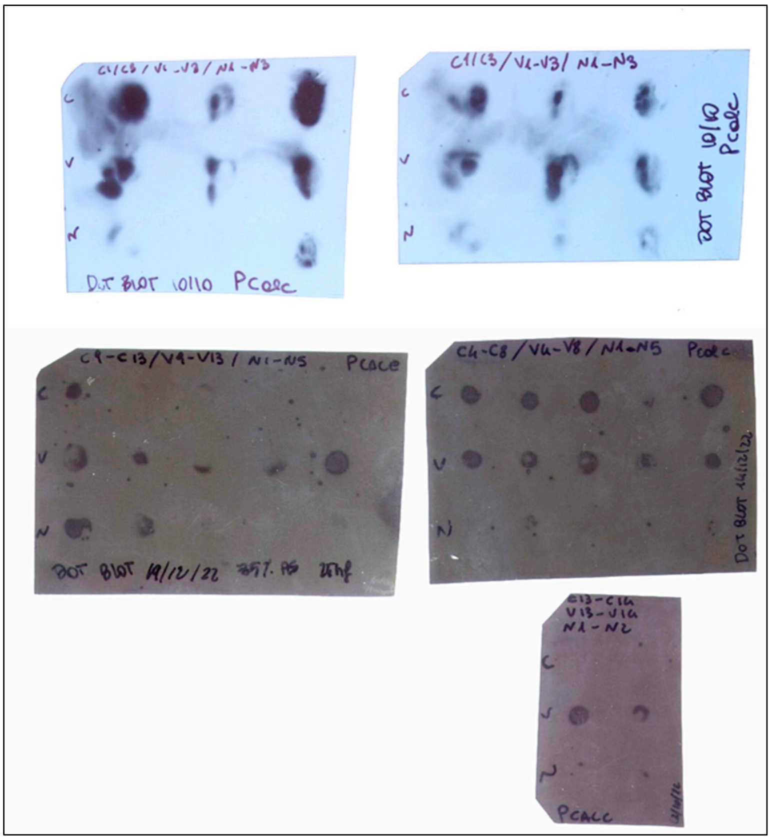

Figure S2. Original blots for Pcalc. The original image corresponding to the figure in the main article is a cropping containing the sample C1-V1-N1.
